# News and Miscellany

**Published:** 1883-10

**Authors:** 


### NEWS AND MISCELLANY.

Personal.—Prof. A. Liautard has been elected Corresponding and Honorary Member of the Societé Véterinaire d'Alsace-Lorraine.

R. H. Harrison, D.V.S., has been appointed by the President and Fellows of Harvard University, instructor in Anatomy in the Veterinary Department of that Institution,

Prof. William Osler, of McGill Medical College, Montreal, has been elected a Fellow of the Royal College of Physicians of London, England. Dr. Osler has held the chair of physiology in the above college for many years, and has done much good work in original investigation. He is at present general secretary of the Canada Medical Association. He is a young man yet, and is to be congratulated on the honor of attaining to the F.R.C.P., solely as the result of his scientific labors.

Dr. Robert Ward, F.R.C.V.S. of England, has been appointed State Veterinarian for Maryland. Dr. Ward brings to the position an extended experience and excellent credentials from those who have known him as a Fellow of the Royal College of Veterinary Surgeons.

A Modest Tribute to Pasteur.—The *Publicateur*, a journal published in the arrondissement of Meaux, contains the following poetical tribute to Pasteur: "M. Pasteur has the glory of being the first to bring knowledge out of mystery and make it irradiate through the Department of Seine-et-Marne; and every sheep which grazes upon the fields sings the glory of the master, and of science!"

Trichinosis from eating Horse-Flesh.—Several Austrian journals report the case of a woman who suffered from trichinosis, caused, it is claimed, by eating horse-flesh. The subject is being investigated.—*Medical Record.*

A gigantic elephant at the Schoenbrunn Imperial Menagerie, near Vienna, was poisoned April 5th by command. Fifty grams of prussic acid were administered to him, after which he died in less than eight minutes.—*Weekly Medical Review.*

The Domestic Animals which annually fall victims to wolves in Russia are valued at about two and a half millions sterling. Besides this loss to the country, there must be taken into account the number of reindeer and other wild animals upon which the wolves prey. Lastly, there is a loss to human life, which cannot be accurately ascertained, but in one year 161 persons were known to have been killed by wolves.

The Superior Orang-Outang Now in London.—The young orang-outang now lodged in the insect house at the Zoo is certainly in point of condition and health, the finest caged specimen ever seen. These creatures usually present a forlorn picture of extreme melancholy, and are generally only too visibly moping away before one's eyes, this even when kept in confinement in the congenial climate of the tropics. Our friend exhibits none of these traces of ill health or sadness, but has full rounded limbs, hair free from a suspicion of manginess, a bright eye, and a rollicking disposition, which can only be appeased by spells of tumbling.

Apart from the fact that this Simian comes from Sumatra where, although first discovered there, the species is far scarcer than in Borneo, it is remarkable that it has shed its teeth, and has acquired a new set. This dental evolution has never before taken place with a large ape in Europe.—*London World.*

BLINKERS.—The question has often been asked, "Why do horses wear blinkers?" We cannot answer the question. It seems to us that they are useless, ugly, and, to some extent, injurious to the eyesight. The most beautiful feature of the horse is its eye. If it were not "hid from our gaze," it would serve to denote sickness, pain, or pleasure. Many a time would a driver spare the whip on seeing the animal's imploring eye. The argument in favor of blinkers is, we believe, that horses are afraid of passing carriages. This objection, if valid, is of little weight, as such timidity would soon be overcome. We trust, now the cruel bearing rein has been cast aside, that blinkers will also be abandoned—a course which would, we feel assured, be attended with advantage to both man and horse.—*Lancet.*

CLOVER BLOAT.—An Iowa man says he has found the following a never-failing cure for clover-bloat: Take a large rope or stick (anything to keep the mouth open), insert it in the mouth, fasten over the horns in order to hold it in place. In fifteen minutes the bloat will have disappeared by the escape of gas from the open mouth. A Michigan farmer says that this peculiar disease, caused by eating green clover when wet with rain or dew, may be prevented by placing a quantity of either hay or straw in the field where stock may have ready access to the same, which they will readily resort to and which will absorb and neutralize the dangerous gases so that no harm will result. He also states that a very effective remedy for stock suffering from such cause is a cold-water bath plentifully applied by pouring over the backs, or where a stream or pool is at hand of sufficient depth to reach the animal's sides, they should be driven in at once, when the bloat will be immediately dissipated. The farmer with bloated cattle may take his choice of cures.—*Chicago Tribune.*

SHOEING A KICKING MULE OR HORSE.—My plan is to tie a three-quarter rope around the pastern of the foot I wish to shoe, then cross it over the back about the withers; run it through the ring of the bit on that side, then through the other ring of the bit; let the man holding the mule or horse grasp the rope bringing it together, and then draw the foot up to the position most convenient for the smith. If the animal wishes to kick, let him try it; but he will quickly find that it will not pay. If he struggles at first, the man holding the rope must not let go, or the work must be done all over again.—*Blacksmith & Wheelwright.*

REMOVAL OF DEAD ANIMALS IN THE CITY OF NEW YORK FOR THE YEAR ENDING MAY 1, 1883.—Horses, 6,354; cows, 187; steers, 21; calves, 816; sheep, 531; cats and dogs, 13,367; mules, 24; goats, 128; hogs, 21; colts, 23; snakes, 2; buffalo, 1; seal, 1; monkeys, 2.

Of the whole number of horses that died in the city in 1882, the Agents of the Society for the Prevention of Cruelty to Animals, killed 2,118.

DISEASED MEAT IN GLASGOW.—Through the imperfect system of meat inspection in Glasgow, the carcases of sixty cows which had died of Anthrax, were sent to the dead meat market in that city and sold for food, having all passed the inspector. As yet, it seems that no fatal results have been known to arise from this unfortunate state of things, still these disclosures cannot but cause a widespread feeling of alarm and indignation among the general public.

THE BOSTON DOG DESTROYER.—During the mad dog scare of 1877, Dr. Watts was appointed a special policeman with power to arrest all vagrants in the shape of dogs found wandering about the city unlicensed or not wearing a collar. During that "dog catching" season, the genial doctor captured and killed 846 dogs found about the streets, belonging to nobody, and which no person would care to own. The success of the doctor's crusade against cur dogs called forth very favorable comments from both officials and citizens, and the next season, 1878, it was considered advisable to continue the work of extermination. That year the total number captured and destroyed was 759, a decrease from the year previous of no inconsiderable nnmber. From that time the work has been continued from year to year by Dr. Watts, resulting as follows: 1879, 719 dogs destroyed; in 1880, 691; in 1881, 418; in 1882, 297. It will thus be seen that there has been a marked decrease in the number captured and killed from year to year, and that last year's "catch" was about one-fourth of the result of the first year. Dr. Watts explains this decrease by the fact that he has made extraordinary efforts toward the capture and extermination of all female cur dogs, and has rid the city of those pests to a very marked extent.

What Shall he do with them.—Dr. Al. Watts, of canine-capturing fame, is now decidedly in a dilemma. The other day he started out upon his annual raid upon friendless curs and succeeded in capturing 16 of them. These were taken to his repository and at the proper time fed upon prussic acid. Death resulted from this diet, and one night 16 cold carcasses lay stretched upon the floor of the repository awaiting the last sad funeral rites. Sixteen little graves had been dug in the fertilizing heaps of the Boston Fertilizer Company upon the dumps at the foot of A street, South Boston, by the sexton; but, alas! the solemn interments in the fertilizer heaps were interfered with by the stern hand of the Boston Board of Health. South Bostonites have long been bewailing the smells from the fertilizer heaps, and, as a slight relief, the board of health forbade the burial of the brutes. The genial doctor sought the assistance of Ward & Co., and other rendering establishments. But no helping hand was extended, for they were all afraid to take the remains because their employés might become poisoned from the prussic acid and perhaps their customers. They would take them if the dogs had been shot, but the doctor thinks that that method of destroying them is too cruel. At present Dr. Watts is in a "box," but it is understood that the board of health has made satisfactory arrangements for the disposal of the dead bodies.

The Cattle Plague.—Statistics show that this terrible disease destroyed over 200,000 head of cattle in Europe between 1869 and 1871. In 1842 it it killed 300,000 head in Egypt, and only died two years later for want of more cattle to destroy; and in 1865–66 it proved fatal to 500,000 head in Great Britain within eighteen months.

In countries where the infected cattle were promptly slaughtered, and all that had been with them promptly disinfected, it has been invariably extinguished at a trifling cost.

In the United States epidemics occurred near Philadelphia in 1834–61; in Louisana in 1837–39, and in Northern New York, in 1825.

By way of prevention it is said that nothing succeeds better than thorough drainage, removal of the animals from dangerous inclosed valleys, rich river bottoms, etc., during the hot and dry season, keeping stock indoors until the dews have disappeared in the mornings, good steady dieting, the maintenance of a healthy action of the organs and skin and a general attention to sound hygienic principles.—*Drovers' Journal.*.

Domestication of the Horse.—M. Cornevin, discussing the earliest evidence of taming the horse, very pertinently sets out with the question, "What is a domestic animal?" and replies, "One that participates in the *domus*, submits itself to the domination of a master, to whom it renders its products or its services, reproduces in captivity, and gives birth to young, which become more and more submissive to control." The idea of domestication comports with that of property in some form. M. Cornevin, for reasons mentioned in his communication, places the time of the event in the bronze age contemporaneous with the bronze bit. The fact seems incontestable that the use of bronze was imported into Europe and Africa from the orient. M. Pietrement, in his work on the origin of the domestic horse, and, before him, M. Pictet, in his *Origines indo-européennes*, have proved that the Ayrans, of the central Asiatic plateau, utilized the horse at a time when Europe was in the stone age. In the discussion which followed M. Cornevin's paper, M. Faure remarked, that while the bronze bit was good proof of the domestication of the horse, the latter may have been tamed long before bronze was known. Indeed, the Gauchos catch the wild horses with a simple lasso. Could not prehistoric man, after catching a horse by means of a lasso, like the Gauchos, have made a simple bridle of raw hide, and have managed the animal thereby ?—*Bull. soc. anthrop. Lyon.*

Maternal Impression.—Dr. T. C. Poole, Mansfield, Texas (*Medical Brief*, June, 1883), reports the following case: On April 17th, his sow gave birth to eight fully developed pigs. Having by accident been present, noticed the ninth as it was expelled; from its peculiar shape and color, curiosity was aroused, and on examination it was found to have the appearance of an elephant. It was destitute of hair, having a proboscis, with dependent ears, two eyes behind upper two thirds of proboscis closely approximated, yet distinct, an abnormal superior maxillary with two or three large teeth, with a long, thin upper lip, with shape and color of an elephant. The sow's gestation lasts three months and twenty days. The impressions were made within five days after conception. On Christmas Day the male was with her; on the 29th of December a menagerie exhibited at our town, had an elephant staked about three hundred yards from lot where the sow was, and in full view. The case is, however, one of atavism, as the pig descended from the proboscidæ, and this was a reversion to its early ancestors. While there is no essential improbability in the maternal impression theory, the explanation of the case as an atavism is much more probable.

Death from Hydrophobia—Fatal Result of the Scratch of a Cat.—Peter J. Byrnes, a lad of 16, died of hydrophobia at Fort Hamilton last month. About six weeks previous Byrnes was scratched by a cat over the left eye and on the back of the neck, and was slightly cut. The cat at the same time scratched a boy named Despard, aged 10 years. Byrnes lived with Mrs. Despard, and was a bright, intelligent youth. The cat was killed, and nothing further was thought of the matter. One evening Byrnes and the boy Despard were playing in a hammock in the yard of the house when Byrnes fell out of the hammock. He immediately became dizzy. He soon recovered, but complained of pains in the throat and jaws, and as night advanced he became delirious and violent, and had to be subdued by force. Dr. De Mund was called in and treated the sufferer for hydrophobia. The next morning Post Surgeon Vollum was called in consultation by Dr. De Mund, and he also pronounced the case one of hydrophobia. Dr. Vollum said that the rumor that hydrophobia could be caused by a cut from the claws of a cat was ridiculous. It was, however, a real case of hydrophobia, and from what he had heard he believed that the cat which attacked the boys had had a fight with a rabid dog, and that he had carried off some of the virus from the dog in his claws.

Blood Poisoning in the Lower Vertebrates.—It is a well-known fact that the teeth of animals which are innocent of any specific poisonous secretion may sometimes inflict an injury distinctly to be classed among "poisoned wounds," resulting in an unhealthy ulcer, for which the mechanical violence offered to the tissues is insufficient to account; or even in fatal contamination of the system. This, of course, arises from the inoculation of decomposed particles of food or other septic matter, adherent to the teeth; and is precisely analogous to the danger of a scratch or cut received in the dissecting-room, or *post-mortem* theatre. It may be mentioned, in passing, that this danger is far greater in conducting an examination of a body within forty-eight hours after death, especially with such as have died of certain inflammatory diseases, than in the ordinary course of dissecting as practised in medical schools, where the "subject" remains on the table for six weeks or two months. That the animals whose bite is liable to these after symptoms should usually be found among the carnivora, is only to be expected; indeed, it has almost passed into a popular proverb that the teeth of a cat, fox, or rat which has fed on carrion are poisonous, though the simple pathology of the circumstances is not generally recognized. Commonly, a badly-inflamed wound, with more or less local complication, is the worst sequence, direct constitutional poisoning from the bite of an animal being rare. It does, however, sometimes come on as a secondary effect, owing to absorption of morbid products from the seat of injury into the circulation. Such creatures as venomous serpents, the heloderme, and those infected with hydrophobia, glanders, farcy, and other communicable diseases, are, of course, excluded from the present consideration.

One would hardly have imagined that a reptile, with its sluggish blood, low vitality, and tolerance of poison and injury, could be susceptible of acute systematic infection from this remote cause. Nevertheless, a case came under my notice the other day at the Zoölogical Gardens, which appeared to me to admit of no other explanation. A grass snake, which had been sent up as a variety on account of the depth and brilliancy of its yellow color, was being fed in a small box with a number of other colubrines, when one of its companions (probably Leptodeira annulata) laid hold of the frog which it had already half swallowed. The robber usually gets the best of it in such a state of affairs, and, having the outer grip, not unfrequently engulfs the legitimate captor as well as the prey. Poor natrix allowed its head to be drawn into the jaws of its despoiler before relinguishing the struggle for its quarry, but let go in time to get away with a scratch on the side of the neck. Shortly afterwards the keeper found it swollen and inflamed as though it had been submitted to the fangs of the hamadryad. The neck continued puffed up and the wound discharged matter for several days, during which the snake manifested great uneasiness. At the end of that time the swelling subsided, but the reptile became apparently paralyzed, and died about ten days after the reception of the injury. I could find no local lesion whatever to account for death.

There is nothing specially remarkable in the fact of a snake inoculating morbific material by its bite, particularly a non-constrictor, which has to depend upon its teeth alone for catching, holding, and slaying its victim, and accordingly buries them deep in the flesh, as the jaws "walk" over it laterally. Moreover, the formation of parts deprives such a creature of the facilities for cleansing its mouth, which most other animals possess. I have experienced one or two painful inflamed and ulcerated sores from the scratches of harmless serpents, possibly—not necessarily—resulting from this cause. It has been shown, too, of late that chemical compounds which may poison the blood when introduced into a vein, are generated in the saliva or mucous of the mouth, even in man, during violent excitement. But, remembering how slowly a reptile is affected by inoculation with the most virulent venom the circumstance of a snake's succumbing under the above conditions to be sufficiently extraordinary to place upon record.—*Field.*

Bequests to the Dick Veterinary College and the University of Edinburgh.—Under the will of Miss Mary Dick, sister of the late Professor Dick, founder of the Edinburgh Veterinary College of that name, it is, we understand, provided, after the payment of £100 to the Society for the Benefit of Widows of Veterinary Surgeons and certain other legacies, that the residue of her estate, with the accumulations of the free income to be derived from the same, shall be held by the trustees of the testatrix until it amounts to £20,000, when it shall be divided into two equal portions, £10,000 being applied in the furtherance of veterinary science in connection with the Veterinary College in Clyde Street, and the other £10,000 in the founding of a Professorship, either of comparative anatomy or of surgical anatomy, whichever of these Chairs her trustees shall consider to be most required in the interest of medical science, in the University of Edinburgh, "in memory of the late Dr. John Barclary and the late Professor John Goodsir," the testatrix adding that she was "led to found this Professorship in memory of these gentlemen, in respect of the great regard that my late brother entertained for them, and that they, I believe, entertained for him." While declaring that the period of accumulation shall not exceed twenty-one years from the date of her death, the testatrix provides that in the event of the £20,000 being reached before the expiry of that period, her trustees shall have full power, after making the division, to continue to hold both or either £10,000 for twenty-one years, and until such an amount is accumulated as may be, in the opinion of her trustees, for the most advantageous promotion of the objects contemplated. In a codicil to her will the testatrix states that she had erected certain houses at the Kirkton, Burntisland, on the property belonging to her brother's trustees, and life-rented by her at a total cost of £800; but she had not then got any title to the ground on which the houses were built; and, in the event of her death before getting such title, and the same thus falling to her late brother's trustees, the sum of £800 more shall be employed by her trustees in the furtherance of comparative anatomy and surgery than in the furtherance of veterinary science. Of the trustees under the will, Professor Turner, Professor Chiene, and Professor M'Kendrick (Glasgow) are declared by the testatrix to have been appointed "in consequence of their special qualifications to carry out" her wishes "in regard to the disposal of the residue" of her estate.—*Scotsman, Aug. 7th,* 1883.

MOORE TOPS FOR STABLE BEDDING.—All who are interested in the care of horses and cattle have long recognized the importance of maintaining the condition of the stables in accordance with the dictates of the highest sanitary rules as regards ventilation, drainage, etc. Bedding especially deserves attention, and the question arises, what material will supply the best, most comfortable, and most economical. Sawdust and tanbark are troublesome and expensive, while straw is cumbersome and a poor absorbent. "Peat-moss" is excellent, but a still better and cheaper substitute for all these is the material furnished by the Litter of Health Co., called "Litter of Health Moore Tops."

CITY HORSE-RAILWAYS.—There are in the United States and Canada 415 street-railways, giving employment to about 85,000 men, 18,000 cars, and 100,000 horses in daily use. These horses consume 150,000 tons of hay, and 111,000,000 bushels of grain. 3,000 miles of track represent an invested capital of $150,000,000. The number of passengers annually carried is 1,212,460,000. In the city of New York there are 110 miles of horse-railway, and 11,866 horses are used to operate them. The horses, together with their harness, expensive lands and stables, feed and grains, make the operating expenses, by including interest, $5,104,596.79 per annum. The average life of the street-car horse in New York is less than three years.

RUBBING THE TAIL.—Rubbing the tail is frequently induced by the presence, within the anus, of a species of intestinal parasite known as ascarides, which are a source of irritation. A simple and efficient remedy for these is salt and water, which may be thrown into the rectum with a syringe; or one ounce of spirits of turpentine, mixed with one pint of linseed oil, may be administered in a similar manner. High feeding, the accumulation of filth, dandruff, vermin, etc., is often the cause of the horse rubbing his tail. The treatment consists in the free use of soap and water, a change of diet to green food, and the application of a wash made of bicarbonate soda, one ounce, water two pints, prussic acid two drs; mix.—*National Live-Stock Journal, Chicago.*

OLD lady to druggist: "I want a box of canine pills." Druggist: "What's the matter with the dog?" Old lady (indignantly): "I want you to know, sir, that my husband is a gentleman!" Druggist puts up some quinine pills in profound silence.—*Druggist.*

THE DISEASES OF MONKEYS.—Mr. J. B. Sutton, in the *Lancet,* August 18th, enumerates the following as causes of death in captive monkeys: Tubercle, bronchitis, pneumonia, empyema, septic pneumonia, rickets, scrofula, typhoid fever, and intussusception; many also suffer from cataract. I have a uterus, taken from a baboon, with acute retroflexion of the fundus associated with atrophy of its anterior wall. Hydatids of the peritoneum were found in two instances, but were not the direct cause of death.

REMARKABLE CURE OF CATARACT IN A DOG.—Dr. E. C. Board writes to the *Lancet:* "For the last nineteen months my little dog (now seventeen years old) has been getting very blind, and a cataract had formed in the right eye, so that she could not see in the least out of it. A few days ago she was playing with or teazing the kittens in the kitchen, when the mother flew at her and scratched her face. The dog gave a scream of pain and ran away, and upon the footman examining her a few minutes after, he found the cataract entirely gone. The eye discharged a good deal for a day or two, but it has quite healed, and the little dog evidently sees as well as ever."

THE STEREOSCOPE IN THE STUDY OF ANATOMY.—SIR: By the permission of M. Channeau, the director of the Lyons Veterinary College, the author of the Comparative Anatomy which is the text book of our veterinary colleges, I performed a series of experiments at Lyons with the object of ascertaining whether the stereoscope could not be used in illustrating anatomical subjects, and thereby much facilitating the study of anatomy.

At the request of the Veterinary Society of the Gironde, Bordeaux, a series of fifty of these stereoscopic views were sent to the veterinary department of the Bordeaux Exhibition, July, 1882.

I enclose the report of this society (taken from the Lyons Veterinary Journal, January, 1883) upon my collection, which I think may prove interesting to your readers. SAM. FRANK ELLIMAN.

Lancaster House, Slough, Feb. 26.

Amongst the most curious of the subjects sent to the Veterinary Society of the Gironde, figures the stereoscope of Mr. Elliman, of Slough.

This stereoscope contains a series of fifty anatomical views of the horse, &c., photographed in the laboratory of anatomy of Professor Arloing, of the Veterinary College at Lyons, winter 1881 and 1882. Everybody knows with what fidelity the stereoscope represents full figures, especially solid bodies with a projecting surface, with a rectangular or polygonial base, as the cube, the pyramid, &c.

Applied to anatomical subjects, the stereoscope causes to stand out in a manner not less remarkable the complicated parts; it prolongs the view to the bottom of the great nervous cavities; it detaches the muscle from the bone which it covers; in short, it recalls to the student, as to the surgeon, the anatomical peculiarities belonging to each region of the body. One might say, to a certain point, that, thanks to this instrument, it is possible to study anatomy in one's own room; that is to say, without operating on the dead body, by the aid of which the conformation of the organs is generally studied. The application of the stereoscope to anatomical studies is, then, a very happy idea, and of which one must allow the Bordeaux Exhibition has given the first example—*Land and Water.*

